# Functional vision in daily life: Clinical and patient-reported outcomes 18 months after enhanced partial range of field IOL implantation

**DOI:** 10.1371/journal.pone.0333174

**Published:** 2025-10-07

**Authors:** Catharina Latz, Annika Licht, Katharina A. Ponto, Johannes Menzel-Severing, David P. Piñero, Alireza Mirshahi

**Affiliations:** 1 Dardenne Eye Hospital, Bonn, Germany; 2 Department of Ophthalmology, University Medical Centre Mainz, Mainz, Germany; 3 Department of Ophthalmology, University of Düsseldorf, Düsseldorf, Germany; 4 Department of Optics, Pharmacology and Anatomy, University of Alicante, Alicante, Spain; University of Warmia, POLAND

## Abstract

**Background:**

Partial range of field (PROF) intraocular lenses (IOLs) provide good distance visual acuity with enhanced intermediate visual acuity. Neuroadaptation to new intraocular lenses can take several months. This study investigates visual and patient-reported outcomes (PROMs) in patients implanted with the PROF IOL ICB00 (Johnson & Johnson Vision) 18 months post-surgery. A particular focus was put on spectacle independence while performing activities of daily life.

**Methods:**

This ambispective, single-center study included 41 patients (aged 48–84). Visual acuities (distance, intermediate), refraction, and PROMs were assessed ≥18 months post-surgery. Spectacle independence was evaluated using the PRSIQ questionnaire. Patients also self-reported on visual quality, task performance, and photic phenomena.

**Results:**

Binocular visual acuity of 0.20 logMAR or better was achieved by 100.0% of patients for uncorrected or corrected distance, by 73.2% for uncorrected intermediate, and by 79.5% for distance-corrected intermediate vision. Photic phenomena were reported by <10%. Mean visual quality scores were 1.68 ± 0.72 for distance and 2.05 ± 0.92 for intermediate vision, where 1 equaled very good and 6 equaled very poor. Complete spectacle independence was reported by 87.8% for distance and 53.7% for intermediate vision. Satisfaction rates were 90.2% (distance), 87.8% (intermediate), and 51.2% (near).

**Conclusions:**

The ICB00 IOL provides excellent long-term distance and satisfactory intermediate vision, with high spectacle independence and even higher patient satisfaction, although most patients require spectacles for near vision.

**Trial registration:**

This study was approved by the medical ethics committee of the Medical Chamber of North-Rhine, Germany (CE # 2124770CE01).

## Background

In 2024, the ESCRS Functional Vision Working Group proposed a functional classification for presbyopia-correcting IOLs depending on two metrics:

(1) the range of field (RoF) achieved in the monocular defocus curve with best correction at distance at 0.2 logMAR visual acuity (VA) level and
(2) the improvement of VA from intermediate to near distance visual acuity (DVA) [1].

**Fig 1:**
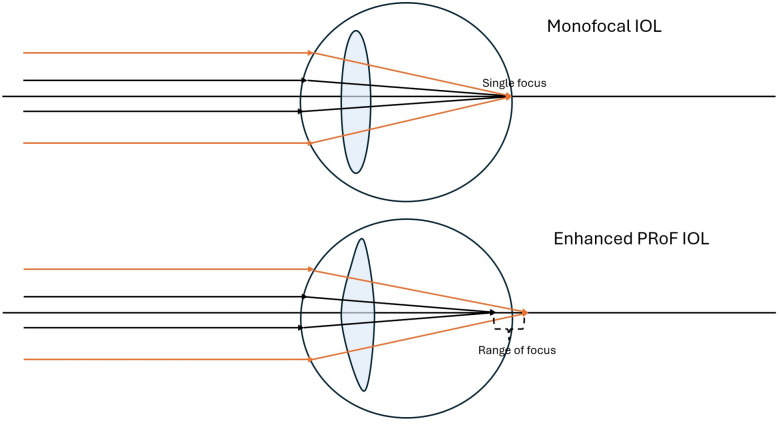
Diagram of conventional monofocal IOL and enhanced partial RoF IOL.

According to this classification the ICB00 (Johnson & Johnson Vision), is an enhanced PROF IOL [2]. This lens features an aspheric anterior and an aspherical posterior surface, with the anterior surface designed to have a continuous increase in power from the periphery to the center [3]. It extends the RoF from distance to > 1.2 to <1.58 diopters (D) (Fig 1). These types of IOLs are also referred as monofocal plus or enhanced monofocal IOLs. A limitation of this functional classification is that it solely describes the VA achieved along the RoF, omitting other significant functional outcomes such as dysphotopsia.

Although excellent DVA and enhanced uncorrected intermediate visual acuity (UIVA), and strong patient acceptance of enhanced monofocal IOLs have been extensively studied [2,4,5], – along with low rates of postoperative photic phenomena comparable to standard monofocal IOLs-, there is limited research on patient-reported outcomes (PROMs) in particular spectacle independence, which reflect patients’ real-world experiences and perceptions [3,6–8].

Given that neuroadaptation and habituation- key factors in patient satisfaction- can take six months or longer, it is particularly important to analyze these at a longer time interval [9]. Morlock et al. identified an important difference in the classification of calling oneself spectacle independent yet using correction for certain activities, which was also related to being able to function better with the correction. These perceived differences were classified as `need´, `wear´, and `function´ and led to the Patient Reported Spectacle Independence Questionnaire (PRSIQ) [10]. To understand spectacle independence and patient satisfaction after ICB00 implantation, visual acuity was tested at different distances, the PRSIQ as well as additional questions regarding photic phenomena and functionality in daily activities were evaluated 18 months after cataract surgery.

## Methods

### Aim, design and setting of the study

This ambispective, non-comparative, single-center study enrolled 41 patients who had undergone sequential, bilateral, uncomplicated cataract surgery with implantation of the ICB00 IOL and had a follow up period of 18 months or longer. Recruitment took place from October 17, 2023, to November 30, 2023, by contacting patients by phone or email. 95 consecutive patients were contacted of which 41 agreed to return to the clinic for additional testing. The last patient was examined on December 08, 2023. Inclusion criteria were corneal astigmatism < 0.75D, age of 45 years or older, and visually significant cataract. Exclusion criteria included systemic diseases that could alter study outcomes, prior ocular or refractive surgery, irregular astigmatism, zonular alterations affecting IOL position or stability, active ocular disease, retinal pathologies and severe glaucoma (defined as a mean deviation deficit > 12 dB on visual fields). Axial length independent of very short or very long eyes was not an exclusion criterion.

Before inclusion, all patients were thoroughly informed about the study´s nature, and written informed consent was obtained in accordance with the tenets of the Declaration of Helsinki. This study was approved by the medical ethics committee of the Medical Chamber of North-Rhine, Germany (CE # 2124770CE01).

### Preoperative examination

The preoperative evaluation included uncorrected distance visual acuity (UDVA) and corrected distance visual acuity (CDVA), objective refraction by autorefractometry, optical biometry and keratometry (IOLMaster 700, Carl Zeiss Meditec), non-contact tonometry, slit lamp biomicroscopy, optical coherence tomography of the macula and optic disc (Carl Zeiss Meditec), and dilated fundus evaluation. The Barrett TK Universal II formula was used for IOL power calculation in all cases with a target of emmetropia, specifically a target value of plano or the first positive value were used to choose the IOL power.

### Long-term follow-up visit

Eighteen months after cataract surgery, all patients were contacted by phone or email and scheduled for a long-term follow-up visit. At this visit, a complete visual evaluation was conducted, including monocular and binocular measurement of UDVA and CDVA, subjective refraction, uncorrected intermediate visual acuity (UIVA) and distance-corrected intermediate visual acuity (DCIVA) (measured at 66 cm). Near visual acuity was not measured. Since EDOF or monofocal plus IOLs tend to produce false negative values on autorefractometry only subjective refraction was used.

Spectacle independence Assessment was evaluated using the PRSIQ [10]. Patients rated the frequency of spectacle need, wear and strain without correction for various activities at near, intermediate and distance on a Likert scale (e.g., always, sometimes, never), enabling the calculation of a PRSIQ score, which is calculated as the average of all individual scores.

Quality of vision and photic phenomena were assessed using a self-developed questionnaire which is depicted in Fig 1. Patients were asked to quantify their distance and intermediate visual quality on a scale from 1 (very good) to 6 (very poor).

Additionally, patients were asked about the perception of halos, glare, blurring, and starbursts at distance and intermediate vision. Daily functionality without spectacles was assessed by asking if the dashboard while driving was clearly visible, and if screenwork and reading were possible without correction. `Yes´ was transposed to 1, `no´ to 6 and modifications like increasing font size to 3, in order to calculate a score similar to the PRSIQ score.

### Surgery

All surgeries were performed by two experienced surgeons using a standard technique of suture-less microincision phacoemulsification [11]. A clear corneal incision with a width of 2.4 mm was manually placed, as were two paracenteses (1.0 mm). Care was taken to achieve a capsulorhexis diameter of approximately 5 mm to ensure complete coverage of the IOL optic with the anterior capsule.

Nuclear disassembly and cortical aspiration were performed using the Centurion vision systems by Alcon (Fort Worth, TX, USA). The IOL was delivered either under irrigation or viscoelastic protection with a shooter.

### Statistical analysis

Data analysis was performed using the software SPSS version 22.0 for Windows (SPSS). Normality of all data distributions was initially evaluated by means of the Kolmogorov-Smirnov test. A descriptive analysis of all continuous variables was carried out, calculating the average values with their corresponding standard deviations and the ranges of maximum and minimum values. For categorical variables, frequencies of different conditions or aspects were determined. For bilateral comparisons, a p-value below 0.05 was considered statistically significant.

## Results

Preoperative patient data are depicted in Table 1. The mean age of the study group was 69.4 years (SD: 9.0, median: 70.0, range: 48–84 years). 24 male patients (58.5%) and 17 female patients (41.5%). were enrolled. Dense cataracts were indicated by a LOCS III score of 4 and 3.5 for nuclear colour and opacity, respectively.

**Table 1:** Preoperative patient data.

Characteristic Median (Range)	Age y	Axial length mm	Visual acuity LogMAR	SE D	LOCS III		
					NO/NC	C	P
**Women,** n = 17 41.5%	70 (50-84)	23.41 (21.51-28.61)	0.2 (0-0.5)	0.69 (−14.5-4.25)	3.5 (0.5-5)	2 (0-5)	1 (0-4)
**Men,** n = 24 58.5%	70.5 (48-82)	24.15 (21.64-26.63)	0.2 (0-1.2)	0.5 (−7.5-6.25)	3.5 (1-5.5)	2 (1-5)	1 (0-6)

D diopters; LOCS Lens Opacification Classification System; NO nuclear opalescence; NC nuclear color; C: cortical; P posterior subcapsular.

**Fig 2:**
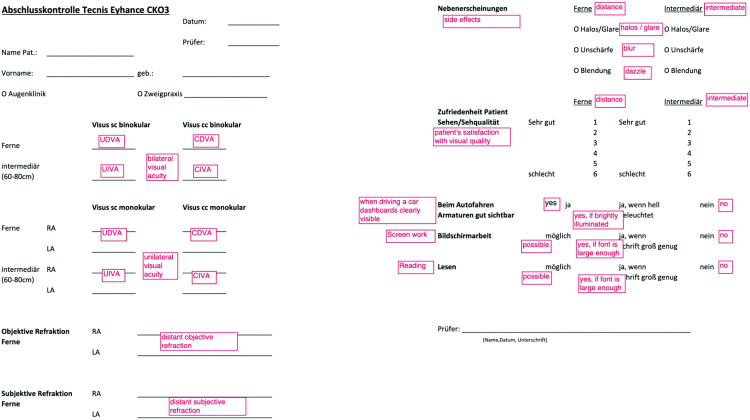
Self-developed questionnaire regarding daily activities. Patients reported visual quality, photic perceptions and daily functionality. Scale from 1 (excellent) to 6 (extremely poor). `Yes´ was transposed to 1, `No´ to 6 and modifications like increasing font size to 3. English translation of prompts in red.

**Fig 3:**
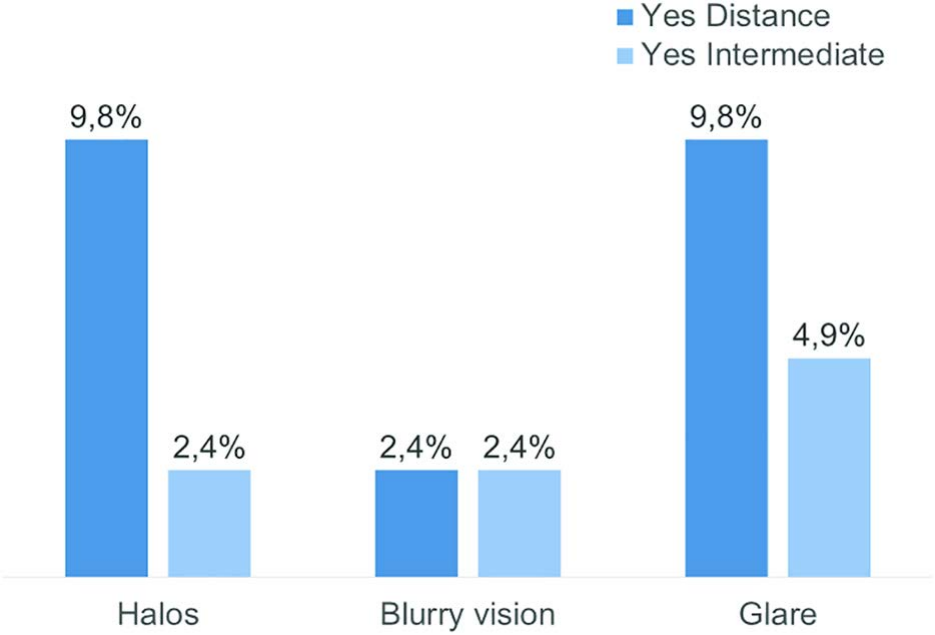
Perception of photic phenomena 18 months postoperatively. Yes, I perceive halos, glare or blurry vision at intermediate or distance vision.

**Fig 4:**
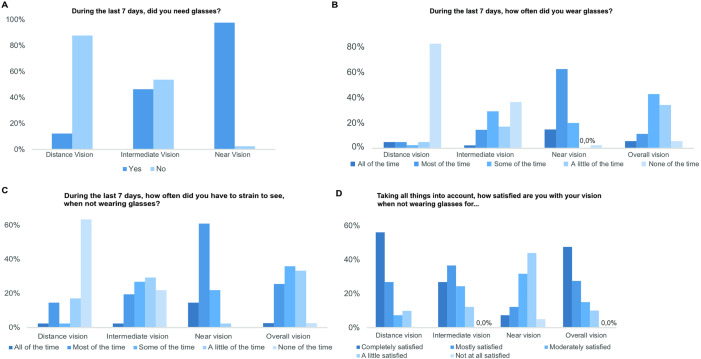
Outcomes obtained with the PRSIQ questionnaire. `Glasses´ were specified as glasses, magnifiers or contacts.

Table 2 summarizes the visual and refractive outcomes 18 months post-surgery: Mean binocular logMAR values were 0.05 ± 0.07 for UDVA, 0.18 ± 0.12 for UIVA, and 0.17 ± 0.11 for DCIVA. Binocular visual acuity of 0.20 logMAR or better was achieved by 100.0% of patients for UDVA or DCVA, by 73.2% for UIVA, and by 79.5% for DCIVA (Fig 1) Postoperative spherical equivalent (SE) was within ±0.50 D in 80.5% of right eyes and 82.9% of left eyes. Likewise, SE was within ±1.00 D in 95.1% of right eyes and 92.7% of left eyes. The PRSIQ and an additional questionnaire were used to assess spectacle independence and satisfaction. The distribution of these answers is depicted in Table 3. At distance, 4 out of 41 (9.8%) patients reported the perception of halos or glare; and 1 out of 41 (2.4%) blurry vision (Fig 2). At intermediate distance these perceptions decreased to 1 in 41 (2.4%) for halos and blurry vision, and to 2 in 41 (4.9%) for glare. Refractive error in this group had a higher spherical equivalent than in the non-halo seeing group, but this difference did not reach statistical significance (Table 4). Patients subjectively graded their visual quality at distance and intermediate vision on a scale from 1 (very good) to 6 (very poor). The mean distance and intermediate visual quality satisfaction scores were 1.68 (SD: 0.72; Median: 2.00; Range: 1–3) and 2.05 (SD: 0.92; Median: 2.00; Range: 1–4), respectively. No patient rated their satisfaction with distance or intermediate visual quality as 4 or worse. (Table 3). 95.1% of patients reported a clearly visible dashboard when driving, increasing to 97.5, when the dashboard was brightly illuminated. 45% of patients were able to perform screen work without spectacles, while 40% had to enlarge the font size. 63.4% of patients achieved spectacle-free reading when the font size was large enough. For distance vision, 87.8% of patients were spectacle free, while 53.7% did not need glasses for intermediate vision (Table 3 and Fig 3A). This was confirmed by the fact, that 82.5% of patients did not wear glasses for distance at any time, while 36.6% did not wear glasses for intermediate vision at any time. (Fig 3B). In contrast, 97.6% of patients required glasses for near vision activities (Fig 3A). In total, 90.2% of patients were completely, mostly, or moderately satisfied with their unaided distance vision. 87.8% were satisfied with intermediate vision, 51.2% with near vision and 90.0% with overall vision (Fig 3D). No adverse events were recorded during the follow-up period, and none of the patients developed posterior capsular opacification (PCO) requiring YAG capsulotomy (Fig 4).

**Table 2:** Summary of long-term postoperative visual and refractive outcomes.

Mean (SD) Median (Range)	Right eye	Left eye	Binocular
**LogMAR UDVA**	0.11 (0.10) 0.10 (0.00 to 0.30)	0.10 (0.09) 0.10 (0.00 to 0.30)	0.05 (0.07) 0.00 (0.00 to 0.20)
**LogMAR CDVA**	0.05 (0.08) 0.00 (0.00 to 0.30)	0.04 (0.05) 0.00 (0.00 to 0.20)	0.02 (0.04) 0.00 (0.00 to 0.10)
**LogMAR UIVA**	0.27 (0.15) 0.30 (0.00 to 0.70)	0.24 (0.14) 0.20 (0.00 to 0.60)	0.18 (0.12) 0.20 (0.00 to 0.50)
**LogMAR DCIVA**	0.22 (0.13) 0.20 (0.00 to 0.50)	0.22 (0.13) 0.20 (0.00 to 0.50)	0.17 (0.11) 0.20 (0.00 to 0.40)
**SE (D)**	0.06 (0.47) 0.00 (−0.75 to 1.13)	0.19 (0.53) 0.25 (−1.00 to 1.50)	---

CDVA, corrected distance visual acuity; DCIVA, distance-corrected intermediate visual acuity; SD, standard deviation; SE, spherical equivalent; UDVA, uncorrected distance visual acuity; UIVA, uncorrected intermediate visual acuity.

**Table 3:** PRSIQ and questionnaire results.

PRSIQ	Text prompt		Response Option						Score
			1	2	3	4	5	6	
1	Need (7 days)	distance	36				5		**1.48**
		intermediate	22				19		**2.85**
		near	1				40		**4.9**
2	Wear (7 days)	distance	33	2	1	2	2		**1.42**
		intermediate	15	7	12	6	1		**2.29**
		near	1		8	25	6		**3.78**
		overall vision	2	12	15	4	2		**2.36**
3	Strain to see when not wearing correction	distance	26	7	1	6	1		**1.76**
		intermediate	9	12	11	8	1		**2.51**
		near		1	9	25	6		**3.88**
		overall vision	1	13	14	10	1		**2.78**
4	Satisfaction with uncorrected vision	distance	23	11	3	4			**1.71**
		intermediate	11	15	10	5			**2.22**
		near	3	5	13	18	2		**3.27**
		overall vision	19	11	6	4			**1.83**
**Self-developed Questionnaire**									
5 **Photic Phenomena**	Halos	distance Intermediate	37					4	**1.49**
			40					1	**1.12**
	Blurriness	distance Intermediate	40					1	**1.12**
			40					1	**1.12**
	Glare	distance Intermediate	37					4	**1.49**
			39					2	**1.24**
6 **Subjective**	Satisfaction Visual Quality	distance	19	16	6				**1.68**
		intermediate	14	13	12	2			**2.05**
7 **Tasks**	Driving	Dashboard visible	39		1			1	**1.17**
	Screenwork	possible	18		16			6	**2.49**
	Reading	possible	6		26			9	**3.37**

Need: No 1, Yes 5; Wear: none of the time 1, a little 2, some 3, most 4, all the time 5; Strain: none of the time 1, a little 2, some 3, most 4, all the time 5; Satisfaction with vision: completely satisfied 1, mostly satisfied 2, moderately satisfied 3, a little satisfied 4, not at all satisfied 5; Photic phenomena No 1, Yes 6, Satisfaction with visual quality: 1–6; Phenomena: no 1, yes 6; Subjective 1–6; Task possible 1, with help 3, no 6

**Table 4:** Refractive error in halo- and non-halo-seeing group.

	SE (D)		P
	Halos		
	Yes		No
Right Eye	0.31 (SD 0.39)	0.03 (SD 0.48)	0.237
Left Eye	0.47 (SD 0.58)	0.16 (SD 0.52)	0.337

D Diopter; SE Spherical Equivalent.

## Discussion

To the best of our knowledge this is the first study to analyze spectacle independence and patient satisfaction at long term after implantation of the ICB00. Our findings regarding excellent distant visual acuity and improved intermediate visual acuity align with those of other studies evaluating the same IOL [3,7,8,12–14]. as well as other models of enhanced monofocal IOLs [15,16]. For instance, Goslings et al^.^ reported mean binocular UDVA of 0.11 ± 0.11, and UIVA of 0.12 ± 0.11 at three months post-implantation [7]. These results are slightly inferior to those observed in our study, which may be attributed to differences in the IOL calculation formula used, though this was not specified in their publication. Similarly, Giglio et al. reported mean postoperative binocular UDVA, UIVA and DCIVA values of −0.03 ± 0.07, 0.17 ± 0.12 and 0.13 ± 0.11, in 30 eyes using the Barrett TK Universal II formula [3]. Mencucci et al. also reported comparable mean binocular UDVA, UIVA and DCIVA values (0.03 ± 0.05 vs. 0.16 ± 0.10 vs. 0.15 ± 0.08) in 80 eyes of 40 patients using the Holladay 1 formula for axial lengths between 22.0 mm and 25.0 mm and Hoffer Q formula for axial lengths < 22.0 mm. Axial length measurements in their study were obtained using the IOL master 500 (Carl Zeiss Meditec AG) [14]. However, other studies have reported worse UDVA and better UIVA values in eyes implanted with the ICB00, particularly when a micro-monovision approach had been chosen or a trend toward a low myopic residual refractive error was observed [17]. Micro monovision is a common strategy to increase the range of field into the near zone, where the dominant eye is targeted towards emmetropia, and the other eye is targeted towards a myopic refractive error. Similarly, with-the-rule astigmatism can lead to a pseudo-accommodation and thereby leading to improved UNVA with enhanced monofocal lenses.

The PRSIQ questionnaire is a validated tool for assessing spectacle independence following cataract surgery [10]. In our study the PRSIQ was complemented with a questionnaire to assess not only satisfaction with uncorrected visual quality, but also ability to perform common intermediate distance tasks such as viewing the dashboard clearly while driving or reading from a computer screen or a handheld device. The grading scale was adapted from the German school system, which is widely recognized and familiar to patients. The scores for visual quality obtained from our questionnaire (1.68 and 2.05) were comparable to those calculated from the PRSIQ (1.71 and 2.22).

Using the PRSIQ, Stodulka and Pracharova investigated a similar IOL with a unique geometry that creates a power gradient from the centre to the periphery [18]. They found that over 80% of patients achieved spectacle independence for distance and intermediate vision with this EDOF IOL.

Similarly, in our study, intermediate vision with the ICB00 achieved scores indicating satisfactory spectacle independence, ranging between 2.05 and 2.85 on both the PRSIQ and the self-developed questionnaire. These outcomes are consistent with results from the validated Catquest 9SF questionnaire used in other studies evaluating the ICB00, where patients consistently perceived a benefit in intermediate vision compared to conventional monofocal IOLs [3,7,8]. Regarding near vision, despite its limitations, approximately half of the patients reported satisfaction with their near visual functionality. This may be attributed to the level of visual acuity provided, which allows for various near-vision activities without spectacle correction. Indeed, 63.4% of patients were able to read without correction if the font size was sufficiently large. This finding is supported by studies by Goslings et al. [7] and Giglio et al. [3], who used the Catquest 9SF questionnaire to assess difficulties in performing vision-related activities after ICB00 implantation and noted improvements in Rasch-calibrated scores for near vision tasks. Lopes et al. demonstrated significant differences between eyes implanted with a conventional monofocal IOL and those with the ICB00, with the latter group reporting less difficulty in tasks such as reading newspaper print and price-tags while shopping [8].

Interestingly, overall satisfaction with uncorrected vision was relatively high with a score of 1.83. This indicates that patients perceive excellent distance vision combined with satisfactory intermediate vision as a highly favourable outcome. This is particularly noteworthy given that our patient cohort included not only previously emmetropic or hyperopic individuals, but also myopic patients, as reflected in the pre-operative refractive errors detailed in Table 1. When correlating axial length and PROMs, there was a trend toward better outcomes in shorter eyes, which was statistically significant when exclusively looking at intermediate vision tasks (S1 File). Given a wide age range from 48 to 84 years in our group, the early presbyopic patients could form a bias in view of higher satisfaction rates and better neuroadaptation.

Photic phenomena were reported by less than 10% of the study group. The refractive error in this group was higher than in the non-reporting group. Since the reporting group was relatively small this difference in refractive error did not reach statistical significance. The fact that the perception of halos and glare decreased with intermediate vision in comparison to distance vision leads in our view to the explanation that a significant reason for the perception of halos and glare is the refractive error. The high number of patients who denied photic phenomena aligns with optical simulations showing that the enhanced monofocal IOL evaluated in this study produces fewer halos compared to other extended range of vision IOLs [19]. Lee and colleagues compared the ICB00 with a diffractive EDOF IOL and found that while spectacle independence was higher in the diffractive group, this came at the expense of more glare and halos [20]. Similarly, Corbelli et al. reported that the enhanced monofocal IOL had the advantage of reduced perception of halos and glare compared to a diffractive IOL [21].

How to measure and rank the effectiveness of presbyopia-correcting IOLs remains a very controversial topic. An important outcome is not only spectacle independence but also side effects such as photic phenomena and the cost of it. Presbyopia-correcting IOLs are more expensive than monofocal IOLs. Since prices vary regionally, it is impossible to give a valid answer to which IOL system offers the best cost-effectiveness ratio. Lan et al. have addressed this question by measuring on 194 patients in two Chinese centres the objective spectacle independence rate – as the proportion of patients with binocular UDVA, UIVA and UVNA all better than 0.1 logMAR-, costs, average cost-effectiveness ratios (ACERs, $/1% rate) [22].

In terms of effectiveness, full RoF IOLs (trifocal (93%) and bifocal (75%) were followed by EDOF with 67.9%. Monovision (14.3%) and monofocal strategies (7.4%) had the least favourable outcomes. In terms of ACERs the EDOF strategy [$72.85/1% rate (95% CI 52.02–93.70)] ranked between the diffractive bifocal [$69.06/1% rate (95% CI 30.89–107.21)], and the trifocal ($93.01/1% rate [95% CI 83.23–102.79]), while monovision [$136.83/1% rate (95% CI – 55.40 to 329.14)] showed less cost effectiveness [22]. Daka et al. have given an overview of seven reviews comparing the effectiveness of different presbyopia correcting IOLs. None of the reports analyzed cost-effectiveness. They conclude, that while spectacle independence in particular for UNVA is best with multifocal IOLs, a mini-monovision approach with nondiffractive EDOF or enhanced monofocal IOLs can be an option for patients concerned about visual disturbances from diffractive designs of trifocal IOLs [23].

Our study has several limitations, including the absence of a control group with a monofocal IOL, the lack of data on corneal spherical aberration in the study patients and post operative contrast sensitivity and defocus curves. We did not measure DCNVA nor UNVA, since the ICB00 is not designed to provide a clear focus in the near range. Additionally, the self-developed questionnaire was not validated through focus groups, cognitive interviews or pretesting, which limits its reliability and validity. However, the similarity on scores between the PRSIQ and the self-developed questionnaire for the question on visual quality serves as a partial validation. Given that neuroadaptation and habituation can take six months or longer [9], this study provides valuable insights into long-term patient-perceived outcomes. As the array of FROF and PROF IOLs continues to expand, long-term data are crucial for improving patient counselling.

In conclusion, the ICB00 provides excellent distance vision and satisfactory intermediate visual quality, resulting in high levels of spectacle independence and excellent long- term patient satisfaction with overall vision. Near visual outcomes, however, varied significantly among subjects and typically required spectacle correction. These findings underscore the ICB00´s potential as a reliable option for patients seeking improved distance. and intermediate vision.

## Supporting information

S1 File Analysis of axial length and PRSIQ and questionnaire-score. P = PRSIQ; Q = questionnaire; intermediate includes all answers relevant to intermediate vision (i.e., dashboard visibility, computer); near includes all answers relevant to near vision (i.e., reading a book).
(TIF)

S2 File Raw data. (TIF)

S3 File Conversion table decimal and LogMAR visual acuity. (TIF)
