# Supplementary figures and images for: Functional vision in daily life: Clinical and patient-reported outcomes 18 months after enhanced partial range of field IOL implantation
Source: PLoS One. 2025 Oct 7;20(10):e0333174. doi: 10.1371/journal.pone.0333174 (PMC12503239; doi:10.1371/journal.pone.0333174)

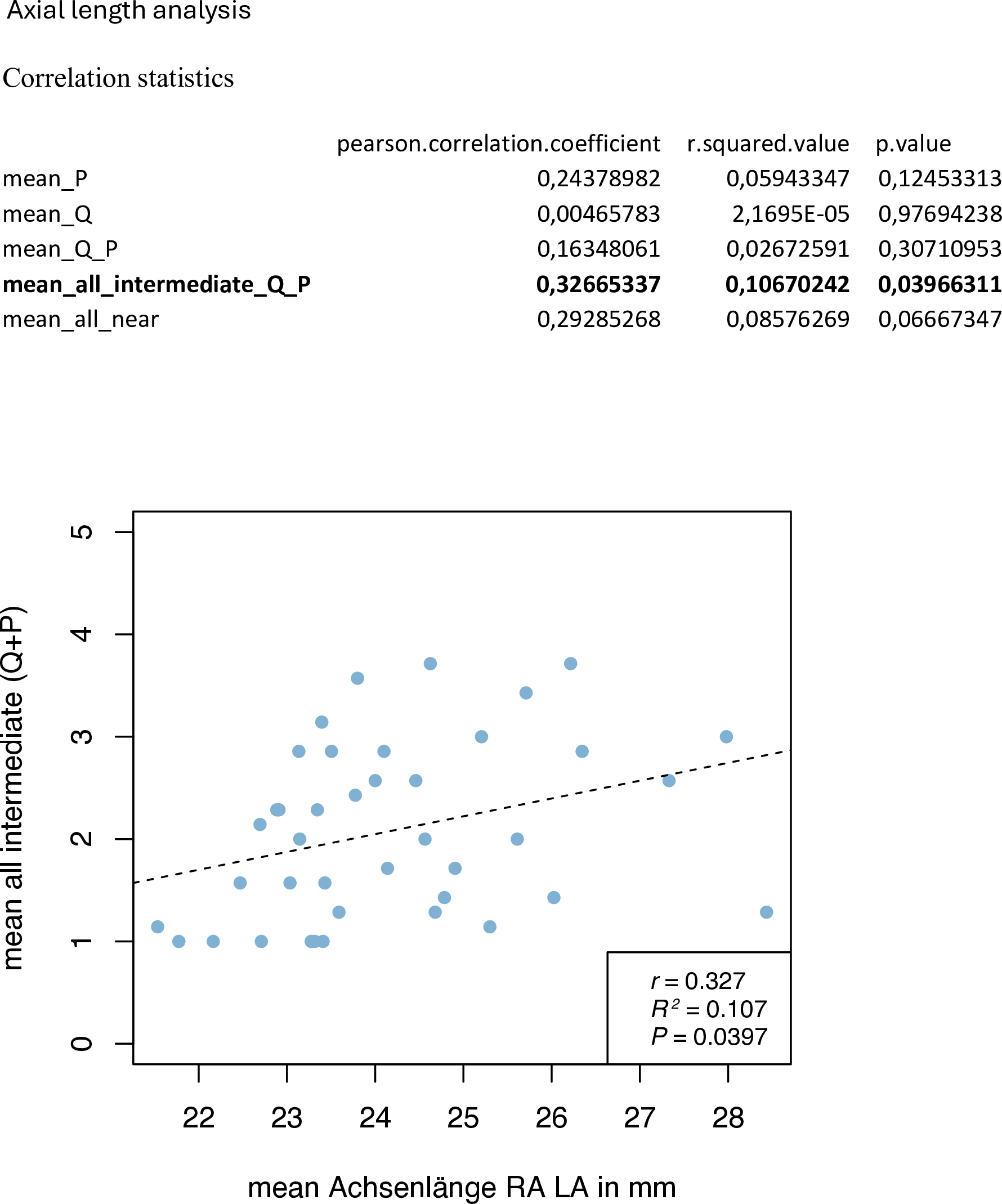

Supplement: S1 File — P = PRSIQ; Q = questionnaire; intermediate includes all answers relevant to intermediate vision (i.e., dashboard visibility, computer); near includes all answers relevant to near vision (i.e., reading a book). (TIF) [file pone.0333174.s001.tif]

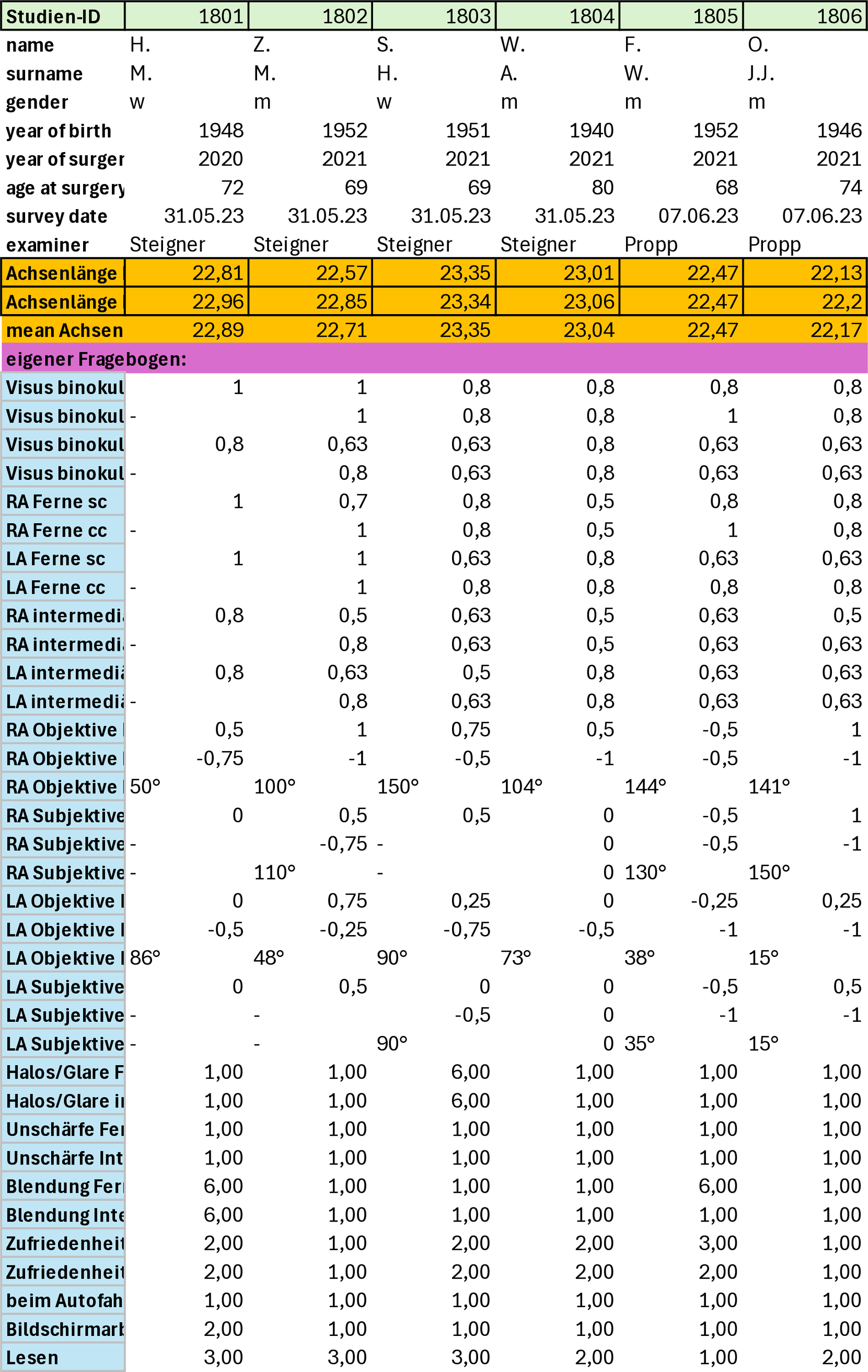

Supplement: S2 File — (TIF) [file pone.0333174.s002.tif]

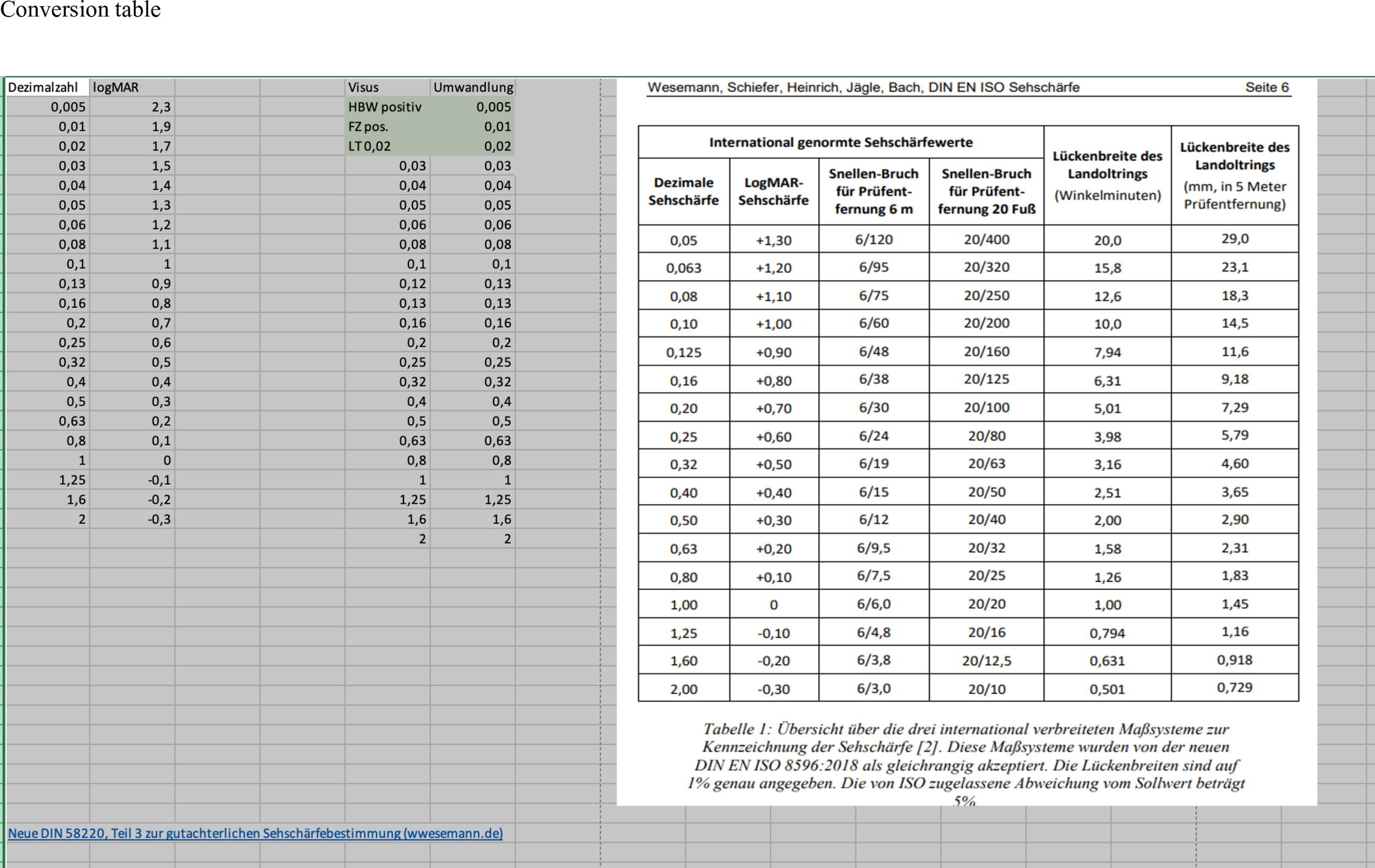

Supplement: S3 File — (TIF) [file pone.0333174.s003.tif]
